# Supplementary material for: Utility of a Molecular Signature for Predicting Recurrence and Progression in Non-Muscle-Invasive Bladder Cancer Patients: Comparison with the EORTC, CUETO and 2021 EAU Risk Groups
Source: Int J Mol Sci. 2022 Nov 21;23(22):14481. doi: 10.3390/ijms232214481 (PMC9696895; doi:10.3390/ijms232214481)
Supplement: Supplementary file 1 [file ijms-23-14481-s001.zip › ijms-2020512-supplementary.pdf]

Supplementary Table S1. Classification of NMIBC samples analyzed by RNA-seq

Samples	Cluster 1 (EP)	Cluster 2 (REC.BCG+)	Cluster 3 (DP.BCG+)	Predicted Cluster
NMIBC1	0.999826468	0.000173532	4.10E-17	1
NMIBC2	1	1.56E-12	7.50E-25	1
NMIBC3	0.999880492	9.65E-17	0.000119508	1
NMIBC4	3.14E-16	1.08E-18	1	3
NMIBC5	0.021204118	0.978795882	1.86E-17	2
NMIBC6	8.93E-23	7.83E-16	1	3
NMIBC7	7.61E-09	2.07E-17	0.999999992	3
NMIBC8	0.999999895	1.05E-07	8.68E-15	1
NMIBC9	0.456925051	0.543074949	5.67E-15	2
NMIBC10	0.99999993	1.51E-18	6.99E-08	1
NMIBC11	0.866265425	1.19E-07	0.133734457	1
NMIBC12	0.816984088	0.183015912	1.99E-12	1
NMIBC13	4.38E-05	5.01E-11	0.999956166	3
NMIBC14	0.841334604	0.158665392	3.66E-09	1
NMIBC15	0.152086245	0.847913755	2.17E-13	2
NMIBC16	3.27E-07	1.77E-17	0.999999673	3
NMIBC17	0.000151326	0.999848674	1.21E-18	2
NMIBC18	5.61E-08	6.07E-17	0.999999944	3
NMIBC19	0.000209725	1.53E-06	0.999788742	3
NMIBC20	0.037962519	6.84E-07	0.962036797	3
NMIBC21	6.50E-13	2.51E-16	1	3
NMIBC22	5.87E-10	0.999999999	1.01E-15	2
NMIBC23	4.18E-06	0.999995823	2.77E-18	2
NMIBC24	0.014531173	0.985123067	0.00034576	2
NMIBC25	0.999767171	0.000232829	1.10E-14	1
NMIBC26	0.014387981	0.985612019	7.33E-16	2
NMIBC27	1.61E-13	4.43E-19	1	3
NMIBC28	8.93E-08	2.81E-07	0.99999963	3
NMIBC29	3.19E-06	0.999996811	5.25E-18	2
NMIBC30	5.11E-08	6.64E-10	0.999999948	3
NMIBC31	1.01E-17	3.33E-18	1	3
NMIBC32	2.11E-06	0.999997893	2.64E-16	2
NMIBC33	5.35E-05	0.999921427	2.50E-05	2
NMIBC34	1.15E-12	4.18E-17	1	3
NMIBC35	6.07E-17	1.43E-17	1	3
NMIBC36	1.41E-07	0.999999858	1.81E-10	2
NMIBC37	1.80E-05	0.999979184	2.84E-06	2
NMIBC38	5.24E-09	0.999999995	7.38E-15	2
NMIBC39	1.15E-12	1.48E-16	1	3
NMIBC40	1.84E-07	0.999999816	6.86E-17	2
NMIBC41	0.951411208	0.048588792	2.07E-17	1

NMIBC42	0.121416476	0.878583524	7.11E-12	2
NMIBC43	2.30E-13	1.30E-16	1	3
NMIBC44	0.998864278	8.29E-17	0.001135722	1
NMIBC45	2.06E-12	8.98E-19	1	3
NMIBC46	0.999999855	1.45E-07	6.28E-12	1
NMIBC47	0.999995848	4.15E-06	2.17E-20	1
NMIBC48	1.22E-10	0.999999999	5.32E-10	2
NMIBC49	0.009803419	0.990196581	5.43E-13	2
